# Construction and Analysis of Immune Infiltration and Competing Endogenous RNA Network in Moyamoya Disease

**DOI:** 10.3390/ijms26167957

**Published:** 2025-08-18

**Authors:** Wenhao Liu, Hanhui Fu, Shiyuan Fang, Jun Ni, Bin Peng

**Affiliations:** 1 Department of Neurology, Peking Union Medical College Hospital, Chinese Academy of Medical Sciences and Peking Union Medical College, Beijing 100730, China; wh-liu16@mails.tsinghua.edu.cn (W.L.); pumchfuhanhui@163.com (H.F.); tinasyfang@outlook.com (S.F.); 2 State Key Laboratory of Complex Severe and Rare Diseases, Peking Union Medical College Hospital, Chinese Academy of Medical Sciences and Peking Union Medical College, No 1, Shuaifuyuan, Dongdan, Dongcheng District, Beijing 100730, China

**Keywords:** Moyamoya disease, immune infiltration, ceRNA network, lncRNA

## Abstract

Moyamoya disease (MMD) is a cerebrovascular condition characterized by progressive stenosis of intracranial arteries, leading to stroke. While MMD was long considered a genetic disorder, emerging evidence suggests autoimmune mechanisms may contribute to its pathogenesis. The role of non-coding RNAs (ncRNAs) in the pathogenesis of MMD is under heated discussion, and a competitive endogenous RNA (ceRNA) network involving MMD-related ncRNAs has not been constructed. In this study, we integrated multiple bioinformatic analyses on transcriptomic data from the middle cerebral arteries of MMD patients and controls. Our analysis revealed a significant enrichment of innate immune system pathways, including antigen processing and macrophage activation, in MMD tissue. We constructed a robust ceRNA network centered on the long non-coding RNA *MALAT1*, identifying 15 core mRNA targets. A classifier built from these *MALAT1*-related genes accurately distinguished MMD patients from controls, with an area under the curve of 0.869 in independent validation. Furthermore, immune deconvolution analysis showed a marked increase in microvascular endothelial cells and a decrease in CD4^+^ memory T cells and regulatory T cells in MMD arteries. The expression of the *MALAT1* network genes strongly correlated with these shifts in cellular composition, positively with endothelial cells and negatively with T cells. Our findings uncover a *MALAT1*-driven ceRNA network that links immune dysregulation to vascular changes in MMD, highlighting *MALAT1* as a potential biomarker and therapeutic target.

## 1. Introduction

Moyamoya disease (MMD) is a cerebrovascular disease characterized by progressive vessel occlusion in the anterior circulation and subsequent strokes [1]. It frequently affects the middle cerebral artery (MCA), leading to reduced blood flow to the brain, collateral vessel formation, and increased risk of transient ischemic attacks, strokes, and hemorrhage [1,2,3]. Since its first description in the 1950s, the etiology of MMD has remained largely unknown. Early histological analyses showed that affected vessels had smooth muscle hyperplasia in the intima and luminal thrombosis instead of atherosclerosis, and no remarkable inflammatory cell infiltration was noticed in the thickened intima of MMD patients [4]. Given that MMD is predominantly found in East Asia and that around 10% of patients have a positive family history, it is considered a genetic disease. However, multiple linkage studies have demonstrated an extremely low penetrance, suggesting that a second hit from environmental factors is required for disease onset. In addition, autoimmune disease seems to be a common comorbidity of Moyamoya vessels [5,6], indicating that immune dysregulation could be an underlying cause. In recent years, a growing body of clinical evidence has linked MMD with autoimmune diseases, such as Graves’ disease and type I diabetes, suggesting that immune dysregulation may be a critical underlying cause [7,8,9]. Furthermore, transcriptomic studies have shown that genes related to the immune response, including antigen processing and cytokine signaling pathways, are upregulated in the arteries of MMD patients [10].

Despite this, the specific molecular mechanisms that link immune activation to vascular remodeling in MMD remain unclear [11]. The role of non-coding RNAs (ncRNAs) in MMD pathogenesis is under heated discussion. Around 90% of the human genome is transcribed into ncRNAs, which play regulatory roles in various molecular pathways, including cell signaling, gene transcription regulation, and epigenetic modification [12,13]. Genes involved in antigen processing and presentation, the cytokine pathway, and the IL-12 pathway are enriched in the MCA of MMD patients; however, the ncRNAs through which these genes are regulated remain undescribed. These ncRNAs and related miRNAs are potential therapeutic targets to prevent progressive vessel occlusion. Among the thousands of lncRNAs, metastasis-associated lung adenocarcinoma transcript 1 (*MALAT1*) has emerged as a crucial regulator in various neurological and vascular diseases [10,14]. In vivo studies have demonstrated that hypoxia, a condition relevant to MMD, upregulates *MALAT1* expression, which in turn induces a phenotypic switch in endothelial cells from proliferative to migratory [15,16]. Silencing *MALAT1* reduces endothelial cell proliferation and migration [17]. Furthermore, *MALAT1* is known to be upregulated following ischemic stroke, where it promotes inflammation [18]. Given its established roles, investigating the network involving *MALAT1* in MMD is a promising direction. That said, a competitive endogenous RNA (ceRNA) network involving MMD-related ncRNAs has not been constructed.

In this study, we aim to compare the gene expression profile in the MCA between MMD patients and healthy controls. We further aim to construct a ceRNA network using the discovered genes. Finally, we conduct an immune infiltration analysis to verify the immunological changes in MMD arteries.

## 2. Results

### 2.1. GSEA Revealed the Involvement of the Innate Immune System

**Figure 1:**
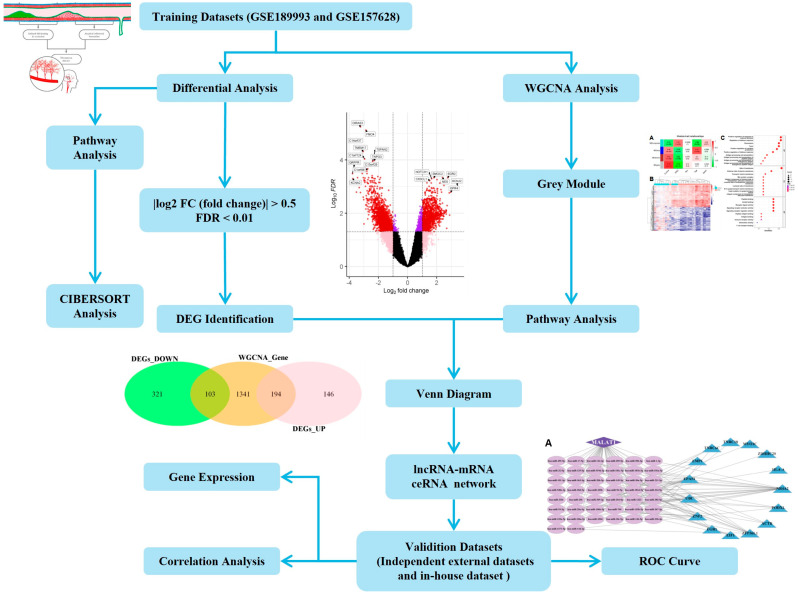
The workflow of this research. Expression profile data were obtained from the middle cerebral artery of Moyamoya disease (MMD) patients. Differential gene expression between patients and healthy controls was examined; meanwhile, weighted correlation network analysis (WGCNA) identified key gene modules for functional annotation further analysis.

**Figure 2:**
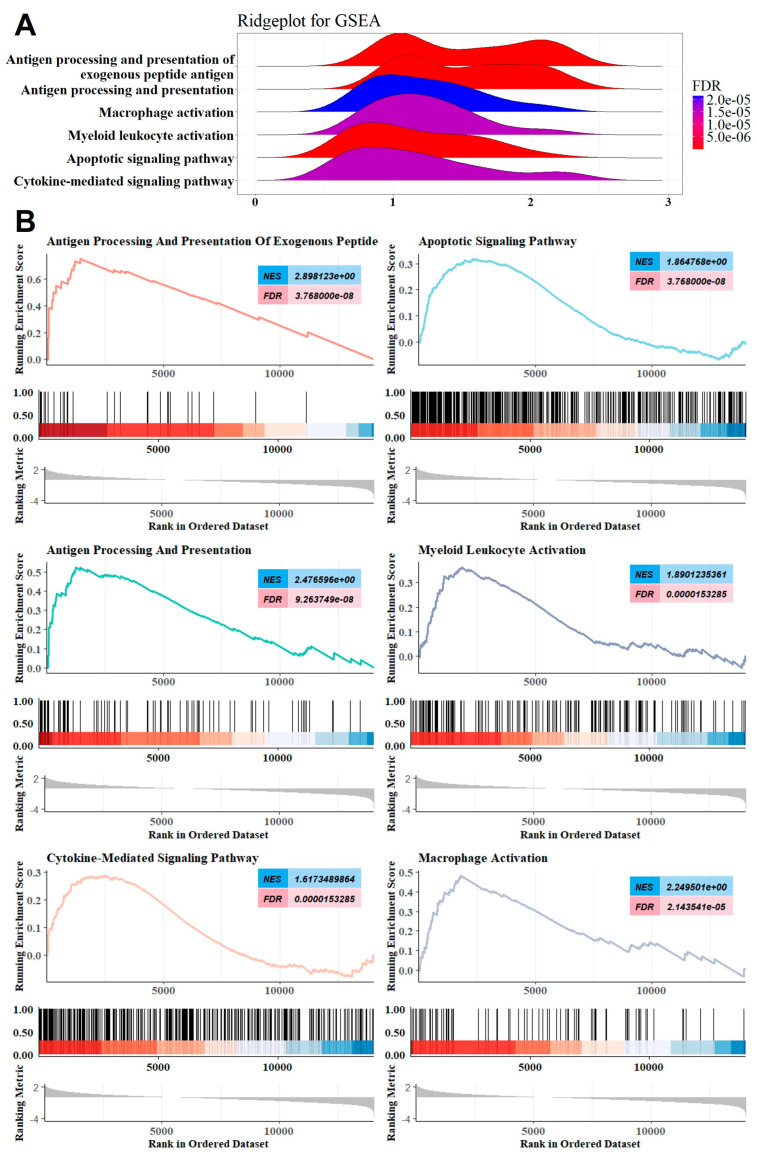
(**A**) Ridge plot showing gene expression distribution in the immune-related annotated gene set. (**B**) Gene set enriched in antigen processing and presentation of exogenous peptide antigens (FDR < 0.001, NES = 2.898), gene set enriched in antigen processing and presentation (FDR < 0.001, NES = 1.865), gene set enriched in macrophage activation (FDR < 0.001, NES = 2.477), gene set enriched in myeloid leukocyte activation (FDR < 0.001, NES = 1.890), gene set enriched in the apoptotic signaling pathway (FDR < 0.001, NES = 1.617), and gene set enriched in the cytokine-mediated signaling pathway (FDR < 0.001, NES = 2.250). Note: FDR means failure detection rate; NES means normalized enrichment score.

The study workflow is presented in Figure 1. First, we created an MMD training dataset consisting of 32 MMD patients and 20 matched controls by merging two MMD datasets (GSE189993 and GSE157628, Table 1 and Table 2). To ensure data consistency, batch effects were controlled, and the different subsets were normalized. The evaluation results showed that data pre-processing was effective and reliable (Figure S2). Next, differential analysis of gene expression was performed when the main clinical information had no significant difference (Table 2). We subjected the logFC value to GSEA, which indicated that the top annotated collection of genes was enriched in the innate immune system (Figure 2A,B).

### 2.2. Co-Expression Modules and GO Analysis

**Figure 3:**
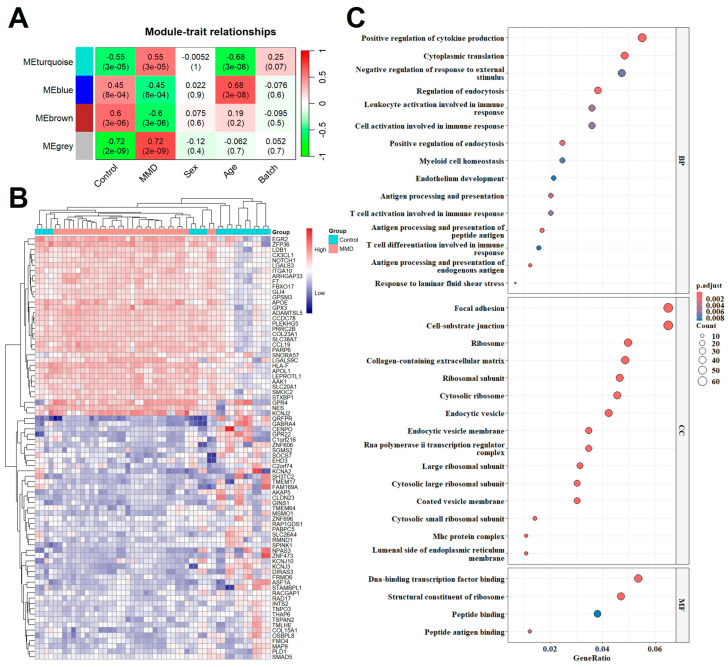
Construction of the weighted co-expression network and module analysis. (**A**) Heatmap of the correlation between MMD susceptibility/persistence and module eigengenes. Each row corresponds to a different module eigengene, and each column corresponds to another MMD trait. Each cell contains the corresponding correlation (first line) and *p* value (second line). (**B**) A clustering analysis based on the grey module eigengenes shown in the heatmap. (**C**) GO enrichment analysis, where the horizontal axis represents the proportion of DEGs under the GO term. Top 10 enriched biological processes associated with the grey module are shown and ordered by gene ratio. BP, biological process; CC, cellular component; MF, molecular function.

Correlation networks are used for identifying clusters of highly correlated genes across microarray samples. We employed WGCNA to construct and analyze MMD-associated networks. We analyzed the merged training datasets, containing 32 MMD and 20 control samples, and selected the optimal β value to match the approximate scale-free topology criterion compared to other datasets. The adjacency matrix constructed based on the criterion of gene distribution conformed to a scale-free network when the soft-threshold power of β = 6 (R^2^ = 0.85) was set, retaining high-connectivity information (Figure S1). In this study, we identified four consensus co-expression modules, among which the grey module shows the closest association between each module and clinical traits (R = 0.79, *p* = 2 × 10^−12^, Figure 3A). We applied a calculated threshold (MM > 0.7 and GS > 0.4) to screen genes closely related to MMD within each module. The heatmap showed that the selected genes were significantly relevant to MMD (Figure 3B). Further dissection of these genes through GO analysis revealed their predilection for involvement in immune-related biological processes and pathways, highlighting the intricate interplay between genetic expression patterns and the immune system in the context of MMD (Figure 3C). Complete list of genes in different Module Clusters can be found in Table S1.

### 2.3. Identification of Overlapping DEGs and GO Analysis

**Figure 4:**
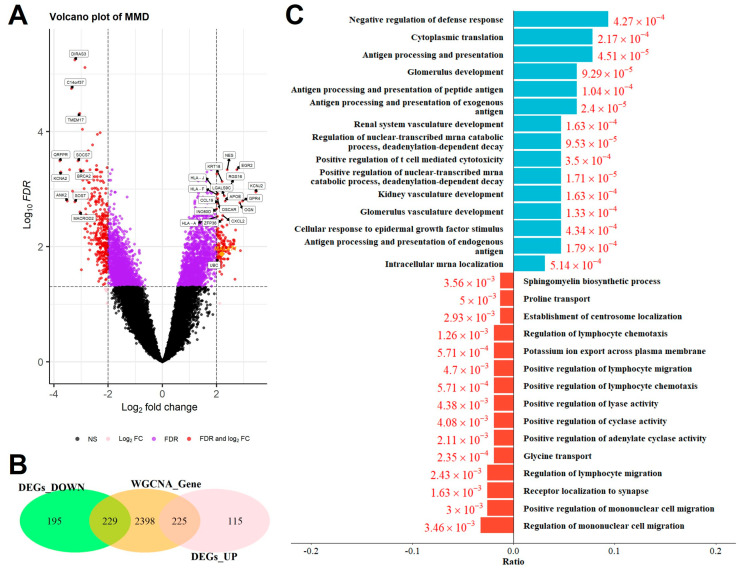
(**A**) Volcano plot shows DEGs in MMD and control samples from the merged GEO dataset. (**B**) Venn diagram of the overlapping genes. (**C**) GO enrichment analysis of the overlapping genes. Top 10 enriched biological processes associated with the upregulated and downregulated overlapping genes are shown.

A total of 764 DEGs for MMD were identified (Figure 4A), and a total of 297 overlapping DEGs were obtained via differential analysis and WGCNA (Figure 4B). In total, 103 downregulated and 194 upregulated overlapping DEGs genes were selected for GO enrichment analysis. Complete result of the above differential expression analysis can be found in Table S2. Under the conditions of an FDR < 0.05, our results demonstrated that upregulated overlapping DEGs are mainly involved in the following pathways: the response to IFN-γ and TNF, cytokine-mediated signaling pathway, cellular response to interleukin-1, and cellular response to EGF stimulus and neutrophil migration. On the other hand, the downregulated overlapping DEGs are mainly involved in the following pathways: leukocyte chemotaxis and migration, positive regulation of GTPase activity, positive regulation of response to external stimulus, and myeloid cell differentiation (Figure 4C).

### 2.4. lncRNA-miRNA-mRNA ceRNA Network Construction

**Figure 5:**
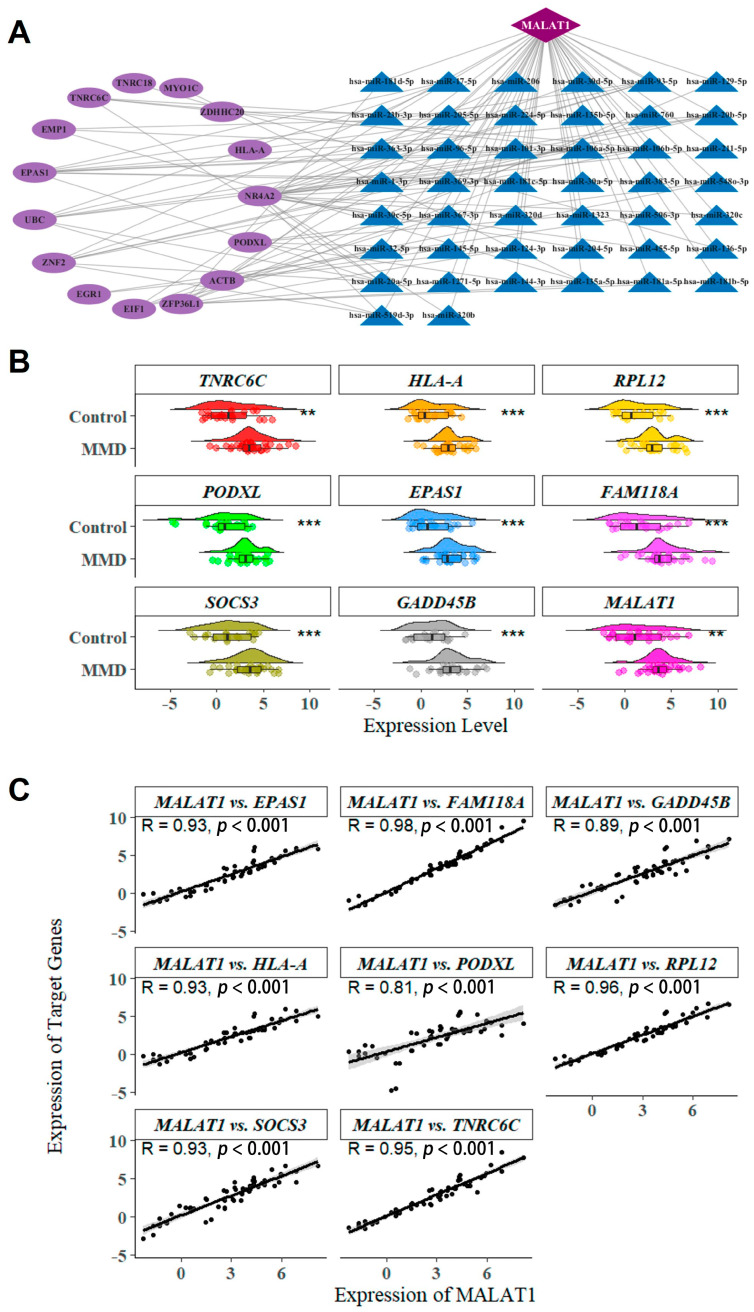
(**A**) MALAT1-related lncRNA-mRNA ceRNA network. Purple color represents lncRNAs, blue color represents protein-coding genes, and pink color represents miRNA. (**B**) The MALAT1-related genes in MMD and control samples from the merged GEO dataset. **: *p* < 0.01; ***: *p* < 0.001. (**C**) Scatter plots and fitting curves of the MALAT1-related genes and MALAT1.

We constructed a ceRNA network from the overlapping DEGs according to lncRNA–mRNA correlation levels and the regulation of experimentally validated miRNA–mRNA/lncRNA in the “multiMiR” package (version: 1.30.0). The ceRNA network links the function of non-coding RNAs with that of protein-coding mRNAs, thus coordinating a number of biological processes, and disruption of the ceRNA network could contribute to disease pathogenesis. This resulted in 1 lncRNA (*MALAT1*), 15 mRNAs, and 44 miRNAs being included in the network (Figure 5A). The lncRNA and mRNAs had differential expression levels in MMD and control samples (Figure 5B), and *MALAT1* and mRNAs except *NR4A2* had a strongly positively correlated expression (R > 0.8, Figure 5C). We further validated the expression and correlation of *MALAT1* and mRNAs using GSE141022 and GSE141024, and the results showed consistency (Figures S3 and S4). Meanwhile, the paired T-test showed a similar miRNA expression change in miR-96-5p (Figure S5).

**Figure 6:**
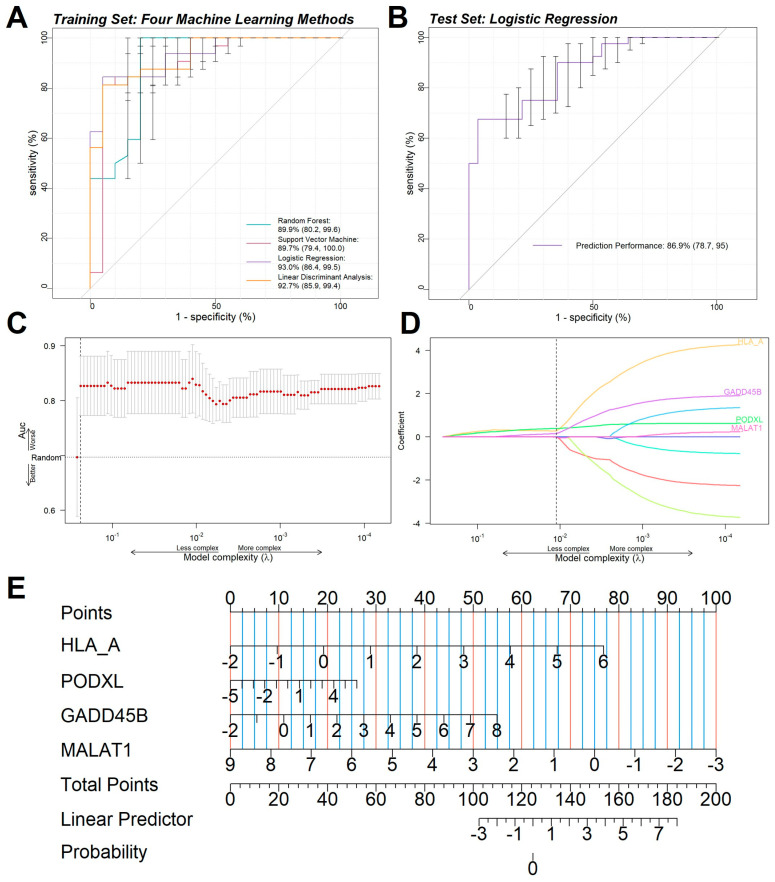
(**A**) The ROC curve of models of MALAT1-related genes using various data-modeling methods (Random Forest, SVM, Logistic Regression, Linear Discriminant Analysis): nomogram. (**B**) The ROC curve of the Logistic Regression diagnosis model. (**C**,**D**) Least Absolute Shrinkage and Selection Operator. Construction of MALAT1-related gene classifier by the LASSO Logistic Regression algorithm shows the process of dimension reduction and coefficient decomposition. (**E**) The predicted scores of different independent predictors are directly proportional to the scale at the top of the nomogram. The sum of the predicted scores of all independent predictors is shown on the scale at the bottom of the nomogram to obtain the susceptibility of MMD.

We reasoned that if *MALAT1* played a crucial role in MMD, the gene expression values of its related ceRNA pairs could discriminate MMD from the control group. Based on five-fold cross-validation, the *MALAT1*-related classifier showed a solid ability to distinguish MMD from controls in the training set, whose AUC value reached up to 0.930 with various machine learning methods (Figure 6A). We then evaluated the logistical regression model’s performance with the independent microarray dataset (GSE141022 and GSE141024). Promisingly, the *MALAT1*-related classifier maintained its prediction capacity, and high AUC values (0.869) were obtained (Figure 6B). To further determine which *MALAT1*-related genes were significantly associated with the biological mechanism of MMD, LASSO regression analysis showed that *PODXL*, *GADD458*, *HLA-A*, and *MALAT1* might play more critical roles in the pathogenesis of MMD (Figure 6C,D). We also utilized Support Vector Machine regression and Random Forest regression to validate our findings (Figure S6). Furthermore, a nomogram was constructed to reflect the contribution of the above gene expression levels to the occurrence of MMD (Figure 6E).

### 2.5. Immune Infiltration Analysis

**Figure 7:**
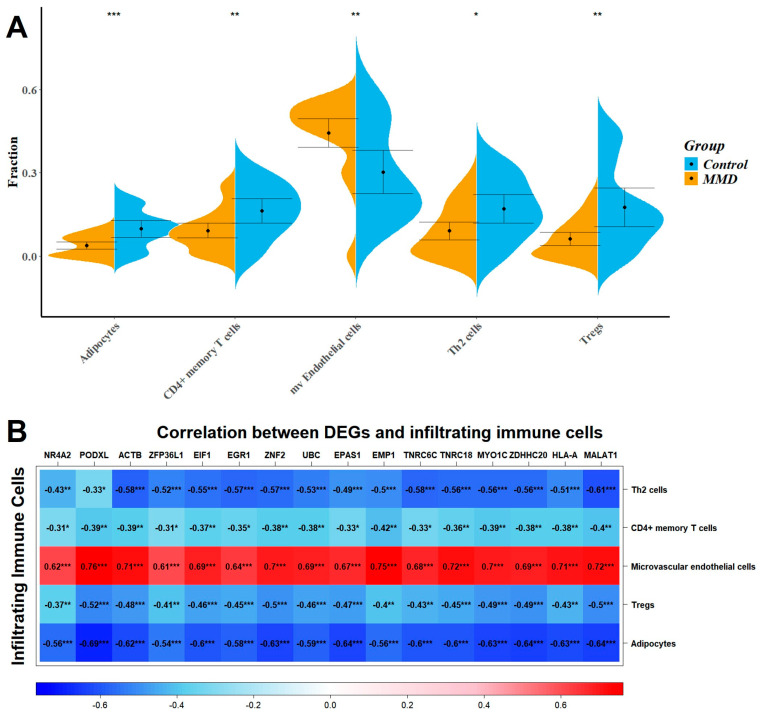
(**A**) Violin diagram showing the significant proportion variation in immune cells obtained using the xCell. *: *p* < 0.05; **: *p* < 0.01; ***: *p* < 0.001. (**B**) Correlation between the gene expression of MALAT1-related genes and immune cells shown in the violin diagram.

To explore the profile of immune cell infiltration, we applied the CIBERSORT classification algorithm to demonstrate changes in the immune cells in MMD. The results of the violin graph showed that the immune infiltration levels of adipocytes, CD4^+^ memory T cells, Th2 cells, and regulatory T cells were significantly downregulated in patients with MMD, while the immune infiltration levels of microvascular endothelial cells were significantly upregulated (Figure 7A). Confirmation of the microvascular endothelial cells’ infiltration levels was achieved through both the MCP-counter and ssGESA tools, yielding consistent results (as illustrated in Figure S6A). Notably, while the MCP-counter tool does not enumerate CD4^+^ memory T cells specifically, the ssGESA tool validated their reduced infiltration, underscoring the consistency of these findings (Figure S6B).

Correlation analysis showed that *MALAT1*-related genes were positively associated with the change in microvascular endothelial cells (R > 0.3, *p* < 0.001) and negatively associated with the change in adipocytes, CD4^+^ memory T cells, Th2 cells, and regulatory T cells (R > 0.3, *p* < 0.001). Our analysis indicated a close relationship between *MALAT1*-related genes and the profile of immune cell variation (Figure 7B). Further validation of this relationship was pursued through application of the MCP-count and ssGESA tools, reinforcing the accuracy of our findings regarding the interaction between *MALAT1*-related genes and immune cell profile alterations (Figure S7).

Pearson’s correlation coefficient was used to analyze the correlation between immune cells (Figure S8). Among them, regulatory T cells were strongly positively correlated with adipocytes (R = 0.766) and CD4^+^ memory T cells (R = 0.518). In addition, microvascular endothelial cells were strongly negatively correlated with adipocytes (R = −0.624), CD4^+^ memory T cells (R = −0.556), and Th2 cells (R = −0.541).

## 3. Discussion

Our study revealed that, compared to control samples, the genes of the innate immune system are highly enriched in the MCA of MMD patients. We further constructed a ceRNA network using *MALAT1* as the key lncRNA. *MALAT1*-related genes were positively associated with the change in microvascular endothelial cells and negatively associated with the shift in CD4^+^ memory T cells, Th2 cells, and regulatory T cells. Overall, our study provides evidence supporting the involvement of dysregulated innate immune activation in the pathogenesis of MMD.

Several clinical studies have linked Moyamoya vessels with autoimmune diseases. A recent study discovered that co-existing Graves’ disease correlated with disease progression and stroke in MMD [7]. A pediatric study reported a 20.3% prevalence of autoimmune diseases in idiopathic MMD, and the most common diseases were thyroid disorders, type I diabetes, and Celiac disease [19]. Similarly, the prevalence in the Asian adult population is as high as 31.0%, with type I diabetes and Graves’ disease being the most common kinds [9,20]. This clinical evidence indicates a potential link between MMD and immune system activation, suggesting environmental and infectious factors could act as a “second hit” to trigger disease onset.

LncRNAs regulate gene expression at transcriptional and posttranscriptional levels and are implicated in cancer and neurological diseases. The precise ceRNA network by which *MALAT1* regulates microvascular endothelial cells remains elusive and may differ among different arteries, as *MALAT1* functions as a microRNA sponge that neutralizes multiple different miRNAs of similar functions [21,22]. Loss of *MALAT1* in ApoE^−/−^ mice precipitates immune-driven atherogenesis, producing ~40% larger aortic-root plaques and 3- to 6-fold elevations in circulating TNF-α, IL-6, and IFN-γ [21]. In isolated mice cardiac microvascular endothelial cells, *MALAT1* acted as a ceRNA for miR-26b-5p. MicroRNA-26p-5b binds *Mfn1* and inhibits its transcription and translation, with *Mfn1* promoting microvascular endothelial cell functions after hypoxia [23]. Animal experiments have shown that miR-96-5p is important for maintaining glutathione levels via various means, which protect the brain from oxidative damage. Our study predicted 17 microRNAs regulated by *MALAT1*; in vivo studies are warranted to determine whether these miRNAs are therapeutic targets to prevent Moyamoya vessel formation.

Our investigation of the immune cell profile in MMD patients yielded compelling findings. We noted an alteration in the cellular composition, with a reduced number of adipocytes, CD4^+^ memory T cells, Th2 cells, and regulatory T cells, while the microvascular endothelial cell population was markedly increased. Adipocytes possess immunomodulatory functions and influence inflammation and immune response [24]. Their decreased infiltration in MMD may suggest a downregulation of certain immune responses, potentially related to disease pathogenesis. CD4^+^ memory T cells, Th2 cells, and regulatory T cells are all critical components of the adaptive immune system. CD4^+^ memory T cells and Th2 cells aid in promoting and coordinating immune responses, while regulatory T cells maintain immune homeostasis and prevent autoimmunity. Their downregulation may cause an immune response imbalance, possibly contributing to MMD pathogenesis.

The upregulation of microvascular endothelial cells in MMD patients is notable. Microvascular endothelial cells are crucial for vascular homeostasis maintenance, and their increased infiltration may reflect a compensatory response to vascular damage. In MMD, internal carotid artery progressive stenosis leads to small-vessel network formation to compensate for reduced blood flow [2]. The increased presence of microvascular endothelial cells might reflect this angiogenic response. Moreover, endothelial cells participate in inflammation and immune responses by producing cytokines and other factors that attract immune cells, contributing to the inflammation in MMD [25].

Correlational analyses underscored a complex interplay among adipocytes, CD4^+^ memory T cells, and regulatory T cells in MMD, suggesting synchronized immune modulation. Strong positive correlations were observed between adipocytes and regulatory T cells (R = 0.766) as well as CD4^+^ memory T cells (R = 0.518). These cells play vital roles in immune modulation, and their association may imply a synchronized immune response in MMD, consistent with previous research on adipocytes’ modulatory influence on T-cell function and the collaborative role of memory and regulatory T cells in maintaining immune balance [26,27]. Conversely, we identified strong negative correlations between microvascular endothelial cells and adipocytes (R = −0.624), CD4^+^ memory T cells (R = −0.556), and Th2 cells (R = −0.541). This suggests a potential dichotomy in the immune response to MMD, where the prevalence of endothelial cells inversely affects the presence of the other immune cells and vice versa. The mechanisms underlying this relationship warrant further exploration. Notably, the increased prevalence of endothelial cells, which are known contributors to inflammation and immune responses, may indicate an inflammatory reaction counteracting the roles of adipocytes, CD4^+^ memory T cells, and Th2 cells.

Our study also indicated that *MALAT1*-related genes were negatively associated with T cells and adipocytes, consistent with previous histologic findings that artery stenosis or occlusion was mainly due to smooth muscle cell proliferation rather than atherosclerosis or macrophage infiltration [4]. Previous studies have established that *MALAT1* promotes inflammation in ischemic stroke, as it is upregulated in neurons and microglia following hypoxia [28,29]. *MALAT1* was also proven to upregulate pro-inflammatory factors in poststroke mice, and *MALAT1* knockdown partially reduced the levels of TNF-α, IL-6, and IL-1β in mouse pheochromocytoma cell lines after oxygen and glucose deprivation/reperfusion [18]. Collectively, this evidence suggests that *MALAT1* primarily promotes inflammation by increasing the expression of pro-inflammatory factors rather than directly causing inflammatory cell migration.

Integrating our results, we can hypothesize a possible mechanism revolving around major signaling pathways. Chronic hypoxia imposed by arterial stenosis in MMD probably upregulates the expression of *MALAT1* in vessels and immunocytes [14,30]. *MALAT1* has been demonstrated to activate the NF-κB signaling pathway, a master controller of inflammation [28,31]. NF-κB activation would immediately result in transcription of pro-inflammatory cytokines [28]. This precisely aligns with our GO analysis identifying significant enrichment in pathways like “response to IFN-γ and TNF” and “cellular response to interleukin-1” as a set of genes augmented in MMD subjects. In fact, *MALAT1* has been demonstrated to elevate levels of important cytokines like TNF-α, IL-6, and IL-1β [28,32,33]. This cytokine-dependent signaling, also recognized in our GSEA findings as a foremost enriched pathway, in turn would propel macrophage activation along with other myeloid leukocytes and affect vascular remodeling in MMD [34]. This encompasses smooth muscle hyperplasia within the intima together with a pro-angiogenic conversion in endothelial cells yielding fragile collateral vessels [34,35,36,37,38]. The *MALAT1*-NF-κB–cytokine signaling platform could therefore be a prime hub uniting immunologic dysregulation with pathological vascular alterations in MMD.

This study has certain limitations. Firstly, it was based on bioinformatic and correlational analyses, which may introduce potential bias owing to differences in microarray platforms, blood collection, RNA extraction, and statistical methods. Secondly, only 32 MMD patients and 20 controls were included; thus, the sample size might be insufficient to identify all lncRNAs with adequate power. Thirdly, the study design is purely in silico. Further in vitro and in vivo studies are needed to test the efficacy of blocking *MALAT1* in preventing collateral circulation formation in MMD.

In conclusion, our study provided evidence of abnormal activation of the innate immune system in MMD arteries and constructed a ceRNA network with *MALAT1* as a miRNA sponge. We further associated *MALAT1* with decreased levels of adipocytes, CD4^+^ memory T cells, Th2 cells, and regulatory T cells. Overall, our study indicates that MMD is an immune-related vasculopathy, and *MALAT1* emerges as a potential key regulator with both anti-inflammatory and pro-migration effects.

## 4. Materials and Methods

### 4.1. Selection and Preparation of Datasets

For this investigation, gene expression profile data were sourced from the Gene Expression Omnibus (GEO) repository (http://www.ncbi.nlm.nih.gov/geo/, accessed on 17 August 2024). The selection process adhered to specific criteria: datasets had to originate from human middle cerebral artery samples, include gene expression profiling, encompass both individuals diagnosed with Moyamoya disease (MMD) and control subjects lacking a history of the condition, and ensure all MMD diagnoses were clinically confirmed according to the Japanese Ministry of Health and Welfare guidelines.

To guarantee data suitability and comprehensiveness, relevant studies were first identified through manual literature searches using targeted keywords. Subsequent data processing and analysis utilized the R programming language (v4.2.2). Following this screening, the GSE189993 and GSE157628 [39] datasets were included and merged as training sets. Potential batch effects arising from merging these datasets were addressed using the comBat function available in the SVA package (v3.38.0) [40]. Next, we normalized the combined datasets and adjusted for covariates using the “Normalizebetweenarray” and “removeBatchEffect” functions in the limma package (v3.54.0). For independent validation purposes, datasets GSE141022 and GSE141024, which also met the established inclusion criteria, were acquired. The RNA expression matrices for all utilized datasets were generated using Agilent’s Feature Extraction Software v11.0.1.1 [41,42,43]. As these datasets derive from microarray technology, not RNA-Seq, the analyses were based on signal intensity values. Table 1 summarizes the included datasets.

### 4.2. Weighted Gene Co-Expression Network Analysis—WGCNA

To explore gene co-expression patterns and identify biologically relevant gene modules without reliance on predefined gene sets, Weighted Gene Co-expression Network Analysis (WGCNA) was employed as an unsupervised clustering method. The analysis focused on the top 20% most variable genes (5000 genes in total) using the “WGCNA” R package (version: 4.2.1) [44]. Scale-free network features were constructed when the power of β was equal to 6 (Figure S1). Genes exhibiting correlated expression profiles were grouped into distinct modules using the dynamic tree cut algorithm. To pinpoint modules most significantly associated with MMD, genes within these modules were filtered based on stringent thresholds for module membership (MM > 0.7) and gene significance (GS > 0.4). This rigorous selection facilitated the identification of genes intimately linked to MMD, enabling a focused subsequent investigation into their potential biological functions and roles in the disease’s pathology.

### 4.3. Identification of Differentially Expressed Genes and Venn Diagram Analysis

Differential expression analysis was performed to identify genes with significantly altered expression levels in middle cerebral artery samples between MMD patients and control subjects. This analysis utilized the limma package, incorporating adjustments for potential confounding variables such as age and sex. Genes were considered differentially expressed (DEGs) if they met the criteria of an absolute log2 fold change (|log2 FC|) greater than 1 and a false discovery rate (FDR) below 0.01. The final set of genes for downstream analysis was determined by identifying the intersection between the DEGs and the genes belonging to the key MMD-associated WGCNA modules, visualized using a Venn diagram.

### 4.4. Biological Function and Pathway Enrichment Analyses

To understand the biological implications of the identified gene set, functional enrichment analyses were conducted. Both Gene Set Enrichment Analysis (GSEA) and Gene Ontology [13] enrichment analysis were performed using the “ClusterProfiler” package (v3.18.1) [13,45]. The GO analysis explored enrichments within the biological process (BP), cellular component [13], and molecular function (MF) categories. For all enrichment analyses mentioned, statistical significance was defined by an FDR less than 0.05. The background gene list used for these functional analyses consisted of all genes detected as expressed in any of the MMD samples included in the training phase.

### 4.5. CeRNA Regulatory Network

A competing endogenous RNA (ceRNA) network, involving long non-coding RNAs (lncRNAs), microRNAs (miRNAs), and messenger RNAs (mRNAs), was constructed based on the hypothesis that lncRNAs regulate mRNA levels by competitively binding to shared miRNAs (acting as miRNA sponges). Potential miRNA-mRNA interactions were predicted using the “multiMiR” package [46]. The miRNA expression levels in 21 human tissues, containing artery tissue, were determined using the TissueAtlas database (current release: July 2022) [47]. Only experimentally validated miRNAs confirmed to be expressed in artery tissue were incorporated into the network construction. Predictions for lncRNA-miRNA interactions were obtained from the Encyclopedia of RNA Interactomes (ENCORI) database (v3.2) [48]. For inclusion in the ceRNA network, lncRNAs needed to have Ensembl IDs listed in the GENCODE database and their interactions supported by evidence from at least one CLIP-seq experiment (clipExpNum ≥ 1) and at least one degradome-seq experiment (degraExpNum ≥ 1) according to the ENCORI [49]. The final ceRNA network, visualized using Cytoscape 3.9.1, included lncRNA-mRNA pairs meeting the following criteria: (1) the lncRNA and mRNA shared at least one common miRNA target; (2) both the miRNA-lncRNA and miRNA-mRNA interactions were experimentally validated; and (3) the shared miRNA was confirmed to be expressed in artery tissue.

### 4.6. Machine Learning and Feature Selection

Utilizing the lncRNA-mRNA pairs identified through the ceRNA network, several machine learning models were developed to evaluate their collective predictive power for MMD status [34]. The algorithms employed included Random Forest, Support Vector Machine (SVM), Logistic Regression (LR), and Linear Discriminant Analysis [34]. RF models were built using the randomForest function from the randomForest package (v4.7-1.1, ntree = 1000). SVM models were implemented via the svm function from the e1071 package (v1.7-12). LR models were constructed using the glm function from the stats package (v4.2.2) with family = binomial(link = logit). LDA was performed using the lda function from the MASS package (v7.3-58.1). Default parameters were generally used, unless otherwise specified. Additionally, the Least Absolute Shrinkage and Selection Operator (LASSO) regression method, implemented through the glmnet package (v4.1-6), was applied to screen for the most influential genes associated with MMD from the input features. These chosen machine learning techniques represent a diverse set of approaches commonly applied in bioinformatics for classification, biomarker identification, and analysis of high-dimensional biological data like gene expression profiles.

### 4.7. Evaluation of Tissue-Infiltrating Immune Cells

To characterize the immune cell composition within the tissue samples based on their gene expression profiles, the xCell deconvolution algorithm was applied via the “immunedeconv” package [50]. Subsequently, Spearman’s correlation analysis was conducted to assess the relationships between the expression levels of the selected MMD-associated genes and the estimated abundances of various immune cell types. Visualizations of these correlations were generated as heatmaps using the ggplot2 package (v3.3.3). Further deconvolution of the expression matrix was performed using the MCP-counter tool, also available within the immunedeconv package, to quantify the abundance of key immune and stromal cell populations. Moreover, single-sample Gene Set Enrichment Analysis (ssGSEA) was employed, utilizing cell-specific gene signatures obtained from the CellMarker 2.0 database (http://117.50.127.228/CellMarker, accessed on 17 August 2024), to provide additional insights into immune cell infiltration patterns.

### 4.8. Extracellular Vesicle Isolation and RNA Extraction

Ethical approval for this study was granted by the Research Ethics Board of Peking Union Medical College Hospital (Approval Number: S-K1914), and all procedures adhered to the principles outlined in the Declaration of Helsinki. Written informed consent was obtained from all participants, who included patients diagnosed with Moyamoya disease (MMD) and healthy control individuals. Further details regarding the participants are available in Table 3. From each participant, 2.5 mL of peripheral blood was collected into K2EDTA-coated Vacutainer^®^ tubes (BD Biosciences, Franklin Lakes, NJ, USA). Subsequently, RNA was extracted from plasma, isolated extracellular vesicles (EVs), and EV-depleted plasma fractions using the exoRNeasy Maxi Kit (Qiagen, Hilden, Germany). RNA concentration was measured using a NanoDrop 2000 spectrophotometer (Thermo Scientific, Waltham, MA, USA), and RNA integrity was assessed using an Agilent 2100 Bioanalyzer (Agilent Technologies, Santa Clara, CA, USA).

### 4.9. MiRNA Sequencing and Bioinformatic Analysis

Small RNA sequencing libraries were prepared based on the TruSeq Small RNA Sample Prep Kit protocol (Illumina, San Diego, CA, USA, Cat No. RS-200-0012), incorporating specific modifications designed to reduce ligation-associated biases. These adjustments included the use of adapters with four random nucleotides at the ends, increased adapter concentrations, a higher polyethylene glycol concentration during ligation steps, and two rounds of size selection following PCR amplification to minimize adapter dimer presence. The quality and concentration of the prepared libraries were validated using the Agilent Bioanalyzer 2100 system with DNA High-Sensitivity Chips (Agilent Technologies, Santa Clara, CA, USA). Sequencing was carried out on the Illumina HiSeq 2500 platform (Illumina, San Diego, CA, USA). Known miRNAs were identified and their expression quantified by aligning reads to the miRbase database using the ACGT101-miR pipeline (LC Sciences, Houston, TX, USA). A specialized normalization method, detailed by Li et al. (2016) [51], was applied to adjust read counts for variations across samples. A miRNA was considered present only if its normalized read count was greater than zero in every sample analyzed. Among the miRNAs identified as significantly altered in MMD artery tissues (17 miRNAs), 3 were also consistently detected across all plasma exosome samples. To specifically analyze MALAT1-associated miRNAs in the plasma exosomes, paired *t*-tests were performed.

## Figures and Tables

**Table 1:** Overview of datasets used in this study. The table provides information about datasets related to Moyamoya disease (MDD) found in the GEO database.

Disease	GEO Accession	Tissue Sources	Data		DEG Count		GEO GPL	Assay Type
			Case	Control	Up	Down		
MDD Train_dataset	GSE189993	MCA	21	11	424	340	GPL16699	Array
MDD Train_dataset	GSE157628	MCA	11	9			GPL16699	Array
MDD Val_dataset	GSE141022	MCA	4	4	567	165	GPL16699	Array
MDD Val_dataset	GSE141024	MCA	4	4	1381	661	GPL16699	Array

Note: GEO = Gene Expression Omnibus; MCA = middle cerebral artery.

**Table 2:** Clinical characteristics of the MMD_Train dataset. The table shows the clinical characteristics of the MMD training dataset, which includes a total of 52 samples, some of which are MMD cases (N = 32), and the others are controls (N = 20).

	Total Sample	MMD, N = 32	Control, N = 20	Statistics/*df*	*p* Value
Gender (% female)	38 (73.08%)	25 (78.13%)	13 (65.00%)	0.51377/1	0.4735
Age, y, mean ± SD	47.4 ± 19.5	43.5 ± 13.7	53.8 ± 25.4	−1.6327/32	0.1124
Atherosclerosis risk factor, mmol/L					
Glucose	105.7 ± 23.3	105.9 ± 23.3	105.3 ± 24.5	−0.11593/37	0.9083
Triglyceride	96.8 ± 63.1	107.8 ± 73.4	75.8 ± 28.9	1.8641/47	0.06863
TC	193.7 ± 39.1	200.0 ± 41.6	181.7 ± 32.2	1.4093/45	0.1744
LDL	118.2 ± 32.7	122.0 ± 36.7	110.9 ± 23.1	0.90712/50	0.3687
HDL	56.5 ± 12.5	58.4 ± 11.6	53.0 ± 13.8	1.7623/44	0.08499

Note: HDL = high-density lipoprotein; LDL = low-density lipoprotein; pt = patient; TC = total cholesterol. Mean values are shown ± SD. The *p* values were calculated using the *t*-test for continuous data.

**Table 3:** Clinical characteristics of the MMD patients and healthy controls from PUMCH. The table shows the clinical characteristics of the serum exosome dataset, which includes a total of 20 paired samples, half of which are MMD cases (N = 10), and the other half are controls (N = 10).

	Total Sample	MMD, N = 10	Control, N = 10	Statistics/*df*	*p* Value
Gender (% female)	12 (60.00%)	6 (60.00%)	6 (60.00%)	0/1	1
Age, y, mean ± SD	38.7 ± 9.1	38.9 ± 9.6	38.4 ± 9.1	0.11936/18	0.9063

Note: PUMCH = Peking Union Medical College Hospital.

## Data Availability

All data supporting the findings of this study are included in the article and its Supplementary Materials. Specifically, the Supplementary Excel file contains: – Supplementary Table S1: Complete List of Genes in Module Clusters; – Supplementary Table S2: Differentially Expressed Genes in Moyamoya Disease. For transparency: the raw data largely come from public databases as described in the paper; the exosome validation component’s raw sequencing reads are currently under confidentiality as part of an ongoing primary study. These raw reads are not required to reproduce the analyses reported here; if needed after the primary study’s publication, de-identified data can be provided by the corresponding author upon reasonable request.

## Supplementary Materials

The following supporting information can be downloaded at https://www.mdpi.com/article/10.3390/ijms26167957/s1.
