# Supplementary material for: Construction and Analysis of Immune Infiltration and Competing Endogenous RNA Network in Moyamoya Disease
Source: Int J Mol Sci. 2025 Aug 18;26(16):7957. doi: 10.3390/ijms26167957 (PMC12386288; doi:10.3390/ijms26167957)
Supplement: Supplementary file 1 [file ijms-26-07957-s001.zip › Supplementary Figures.pdf]

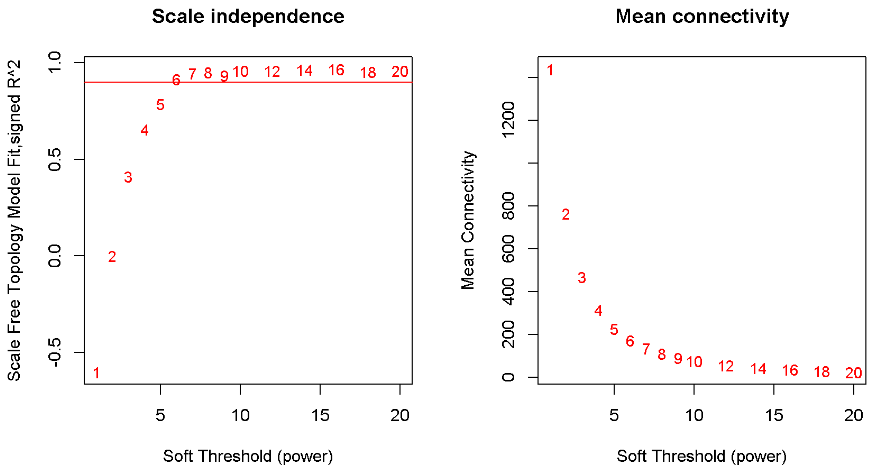

Supplementary Figure S1. Soft threshold power screening and scale-free network construction.

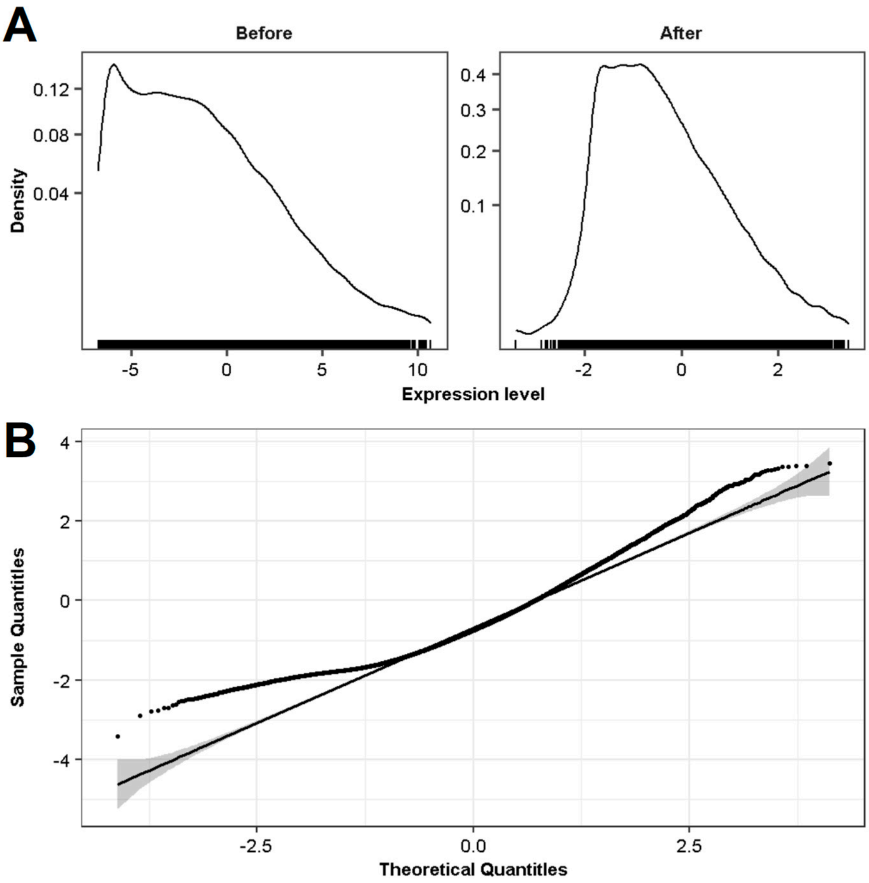

Supplementary Figure S2. The correction of datasets used in this study. (A) The density plots of the merged MMD dataset before (left) and after (right) normalization and removing batch-effects. (B) The Q-Q plot of the merged dataset after correction.

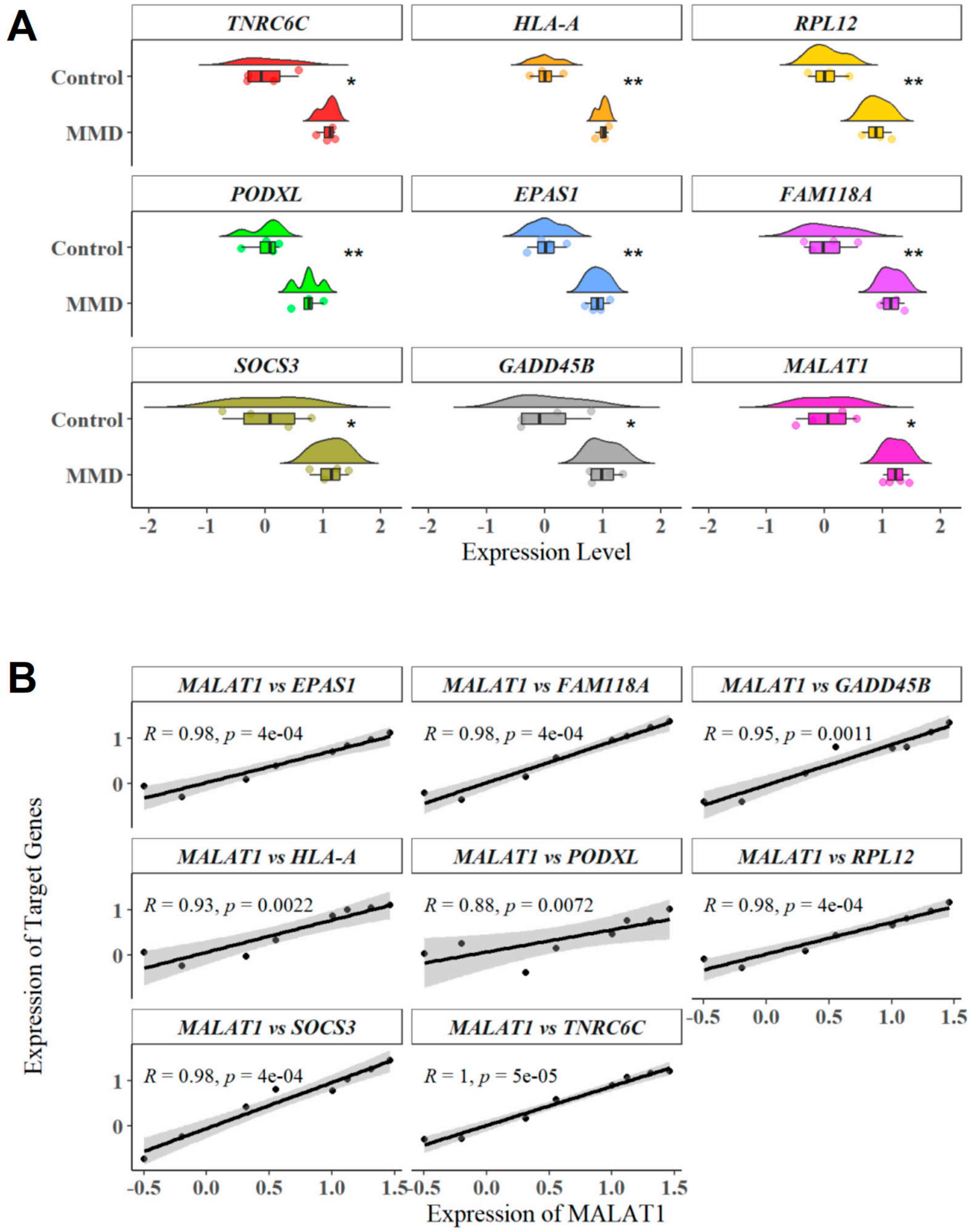

Supplementary Figure S3. (**A**) The MALAT1-related genes between MMD and control samples from the GSE141022 dataset. (**B**) Scatter plots and the fitting curves of the *MALAT1*-related genes and *MALAT1*.

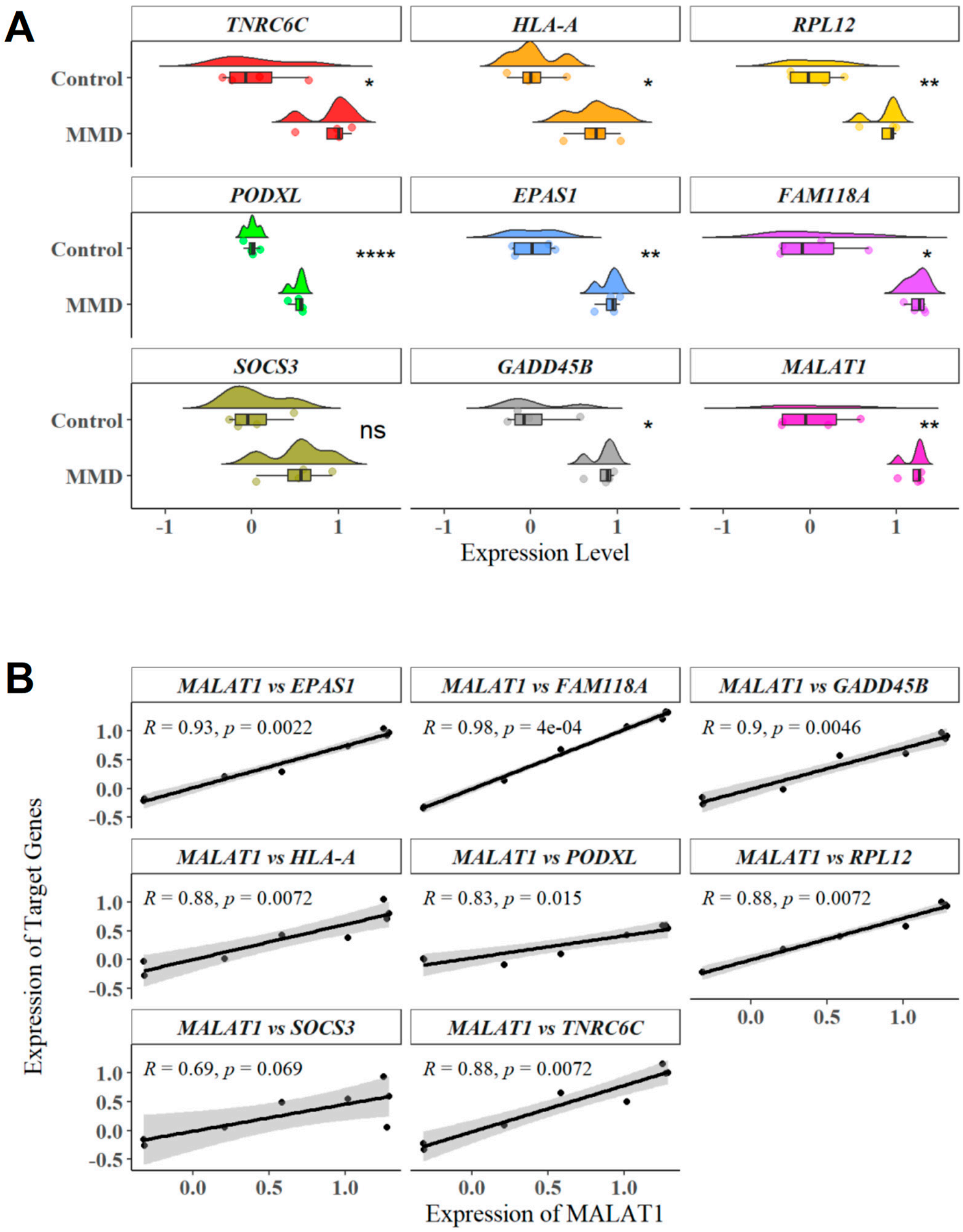

Supplementary Figure S4. (**A**) The *MALAT1*-related genes between MMD and control samples from the GSE141024 dataset. (**B**) Scatter plots and the fitting curves of the *MALAT1*-related genes and *MALAT1*.

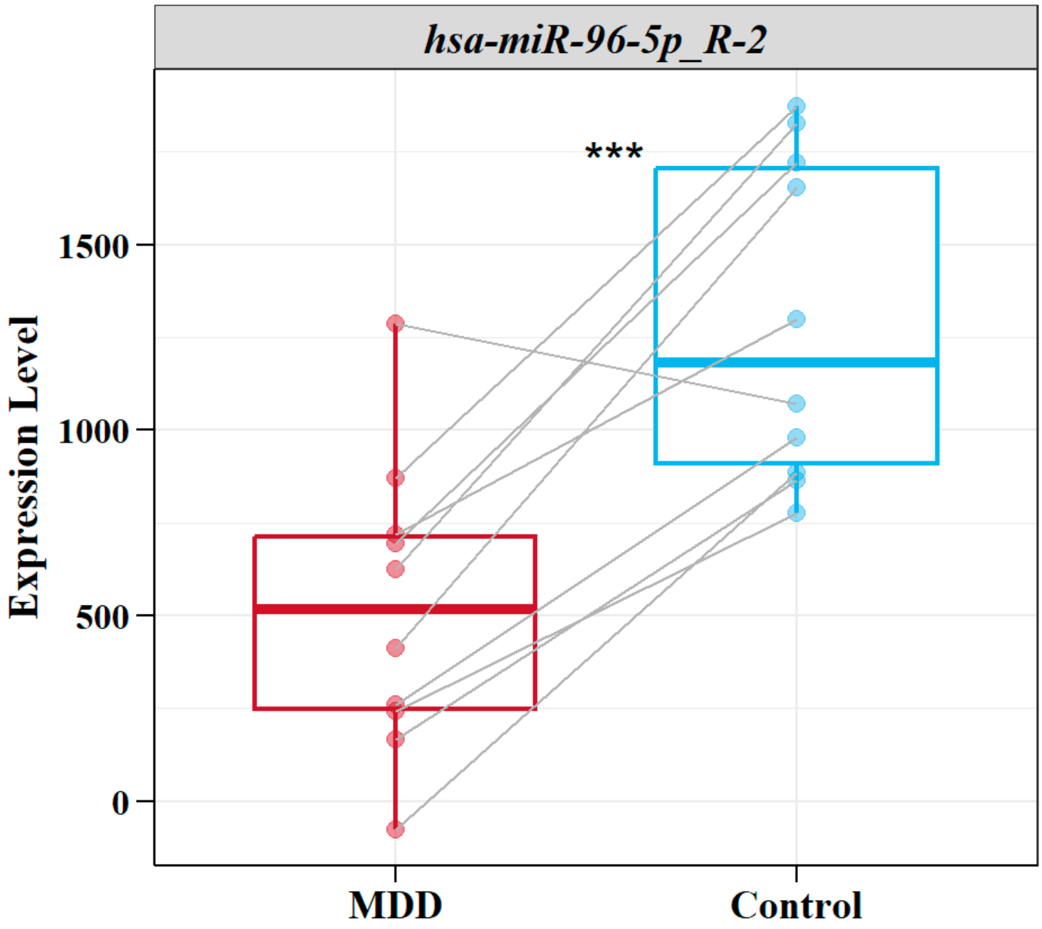

Supplementary Figure S5. The *hsa-miR-96-5p_R-2* expression by miRNA sequencing on serum exosomes from MMD patients and healthy control. Boxplot showing the significant down-regulation of *hsa-miR-96-5p_R-2* in patients with Moyamoya disease via Paired T-test. ***:P < 0.001

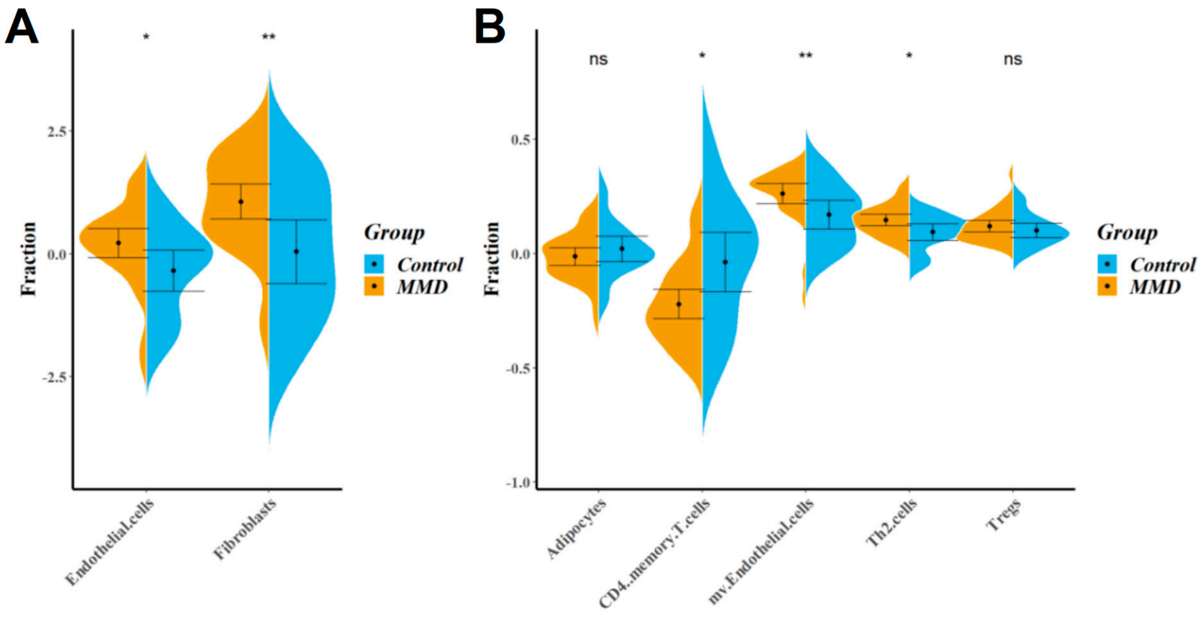

Supplementary Figure S6. (**A**) Violin diagram showing the significant proportion variation of immune cells obtained using the MCP-counter tool. *:P < 0.05; **:P < 0.01 (**B**) Violin diagram showing the significant proportion variation of immune cells obtained using the ssGSEA tool. ns: Nonsense; *:P < 0.05; **:P < 0.01.

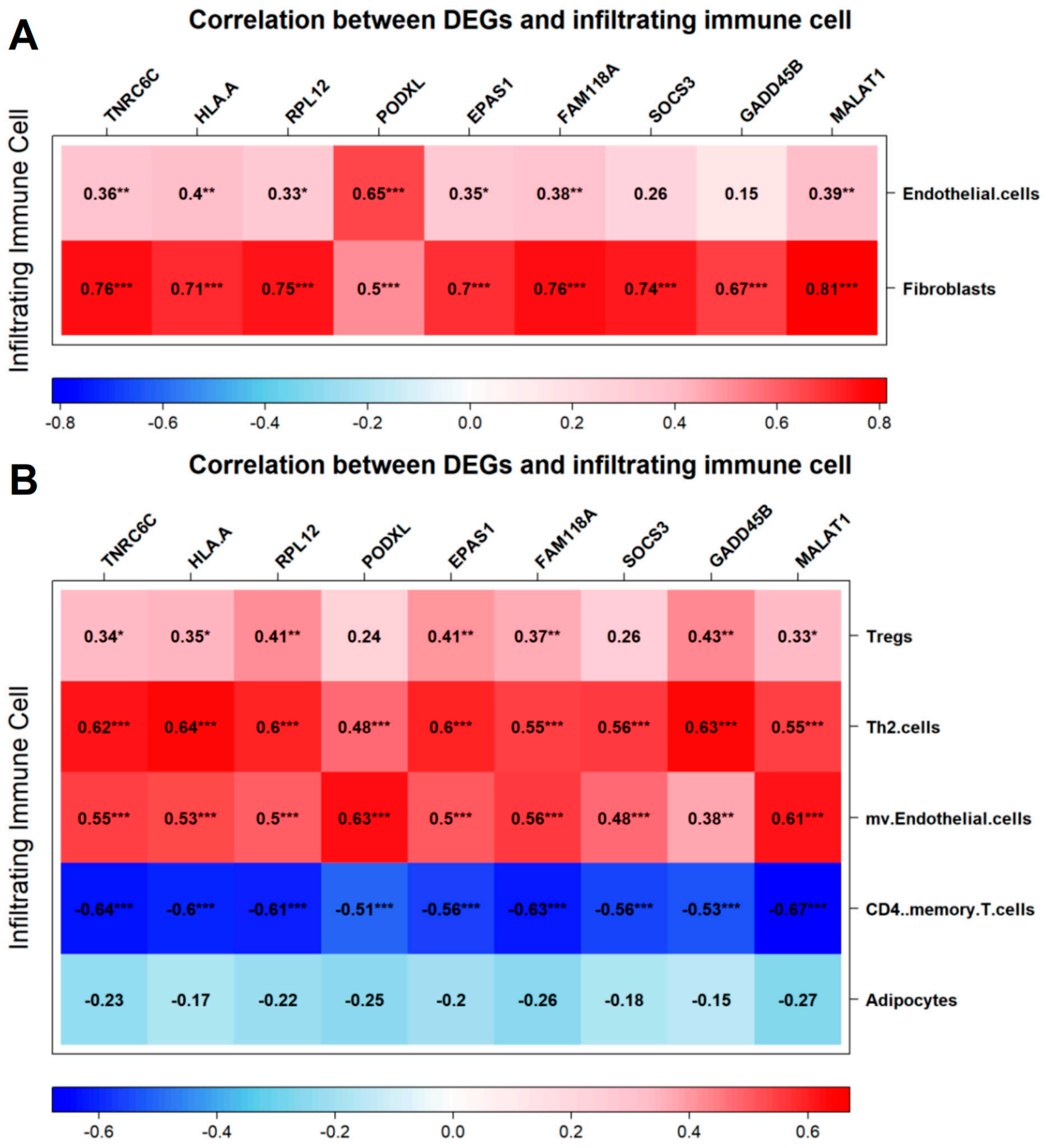

Supplementary Figure S7. (**A**) Correlation between the gene expression of *MALAT1*-related genes and immune cells derived from the MCP-counter tool shown in the violin diagram. (**B**) Correlation between the gene expression of *MALAT1*-related genes and immune cells derived from the ssGSEA tool shown in the violin diagram.

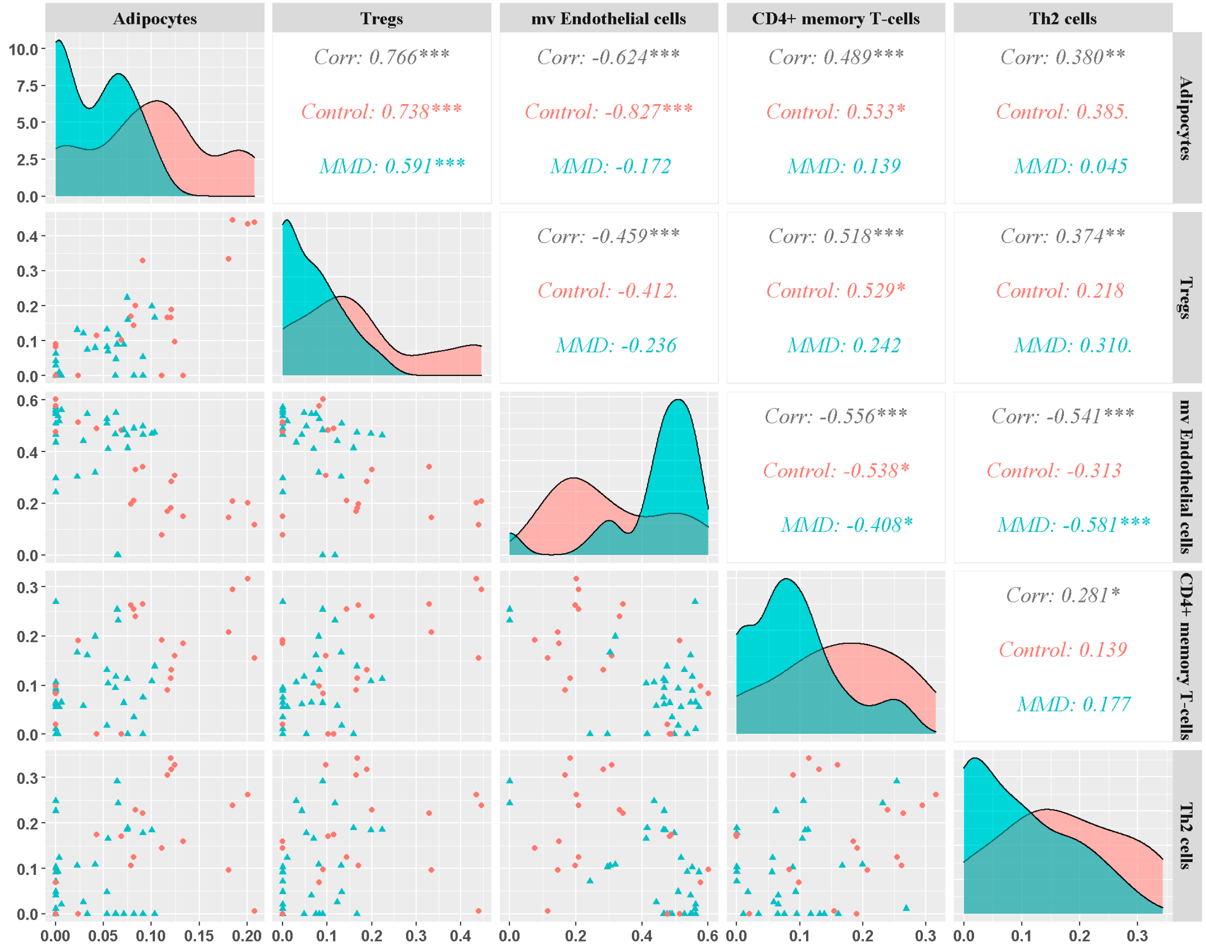

Supplementary Figure S8. Correlation between the proportion variation of immune cells.
